# Totally endoscopic aortic valve replacement with concomitant trans-aortic mitral valve repair for mitral regurgitation

**DOI:** 10.1186/s13019-021-01694-6

**Published:** 2021-10-30

**Authors:** Antonios Pitsis, Nikolaos Tsotsolis, Harisios Boudoulas, Konstantinos Dean Boudoulas

**Affiliations:** 1 Thessaloniki Heart Institute, European Interbalkan Medical Center, 57001 Thessaloniki, Greece; 2 Division of Cardiovascular Medicine, The Ohio State University, Columbus, OH USA

**Keywords:** Minimally invasive cardiac surgery, Totally endoscopic aortic valve replacement, Totally endoscopic mitral valve repair, Edge-to-edge repair, Trans-aortic mitral valve repair

## Abstract

**Background:**

Minimally invasive aortic valve procedures through a hemi-sternotomy or a right anterior mini-thoracotomy have gained popularity over the last several years. Totally endoscopic aortic valve replacement (TEAVR) is an innovative and a less invasive (incision-wise) surgical aortic valve replacement technique. The operative steps of TEAVR have been reported previously from our group. Mitral regurgitation (MR) frequently accompanies aortic valve disease that at times may also require repair. Totally endoscopic surgery in such cases has not been tested.

**Presentation of the technique:**

We present a surgical technique for a totally endoscopic approach to aortic valve replacement and concomitant mitral valve repair for primary and secondary MR. An aortotomy incision was used avoiding an atriotomy, which results in an increase in cross-clamp (XC) and cardiopulmonary bypass (CPB) times that could be associated with higher mortality and morbidity. Neochords (artificial chordae tendineae) were used for primary MR and an edge-to-edge approach for secondary MR.

**Conclusion:**

TEAVR and concomitant mitral valve repair can be performed successfully with reasonable XC and CPB times with excellent short-term results.

**Supplementary Information:**

The online version contains supplementary material available at 10.1186/s13019-021-01694-6.

## Introduction

While endoscopic mitral and tricuspid valve procedures have become a first line treatment in many centers globally, this is not the case for the aortic valve. Minimally invasive aortic valve replacement or repair is usually performed through a hemi-sternotomy or right anterior mini-thoracotomy. The operative steps of totally endoscopic aortic valve replacement (TEAVR) that has been routinely used at Thessaloniki Heart Institute, Thessaloniki, Greece, are described; more than 200 cases have been performed over a 2 year period7 [1].

TEAVR is a less invasive approach (from an incisions stand-point) associated with shorter intensive care time and hospital length-of-stay. This method allows patients to return to work sooner compared to traditional surgery; however, it is associated with prolonged cross-clamp (XC) and cardiopulmonary bypass (CPB) times, particularly during the learning curve of this approach, albeit steep [1]. Primary or secondary MR (approximately 1–3% and 5–10%, respectively) may be associated with aortic valve disease, particularly in patients with bicuspid aortic valves or in patients with coronary artery disease. In these cases, mitral valve repair can be performed via a trans-aortic approach simultaneously with TEAVR, thus avoiding an atriotomy that would result in an increase in XC and CPB times.

We present our technique of TEAVR and concomitant trans-aortic mitral valve repair for primary and secondary MR. In primary MR (Additional file 1: Video S1), artificial chordae tendineae are used to repair the prolapsing/flail mitral valve in which the length is calculated preoperatively by transesophageal echocardiography, as previously reported [2]. In secondary MR (Additional file 2: Video S2), the edge-to-edge repair method is used [3].

Written informed consents for publication of their clinical details and clinical images were obtained from the patients. Copies of the consent forms are available for review by the Editor of the journal.

## Presentation of the technique

This endoscopic approach is performed through a 3 cm right parasternal working incision in the 2nd intercostal space (ICS) and a 10 mm port for the 3D 30 degrees Karl Storz endoscope in the same ICS laterally, anterior to the right anterior axillary line [1]. An extra-extra small Alexis soft tissue protector is deployed through the incision. On full CPB from the groin, the Chitwood clamp is inserted through a separate stab wound incision (3 mm) in the 1st ICS, cephalad to the port of the endoscope, and Custodiol cold crystalloid cardioplegia is given in the aortic root, or directly to the coronary ostia in cases of aortic regurgitation. After the heart is arrested, a right superior pulmonary vein vent is inserted through a separate stab wound incision (3 mm) in the 5th ICS, anterior axillary line. A transverse aortotomy is opened proximal to the fat body of the aorta (3 cm distal to the right coronary artery), the diseased aortic valve is excised and the annulus is debrided and washed. A metal net spreader (Fehling Instruments, DE) is inserted inside the aortic annulus to facilitate exposure of the mitral leaflets. The use of the metal net is of paramount importance for the exposure of the mitral leaflets and sub-valvar apparatus; its use is essential to freeze the aortic annulus open, because operation on the mitral valve cannot be performed when the aortic annulus is collapsed.

**Fig. 1:**
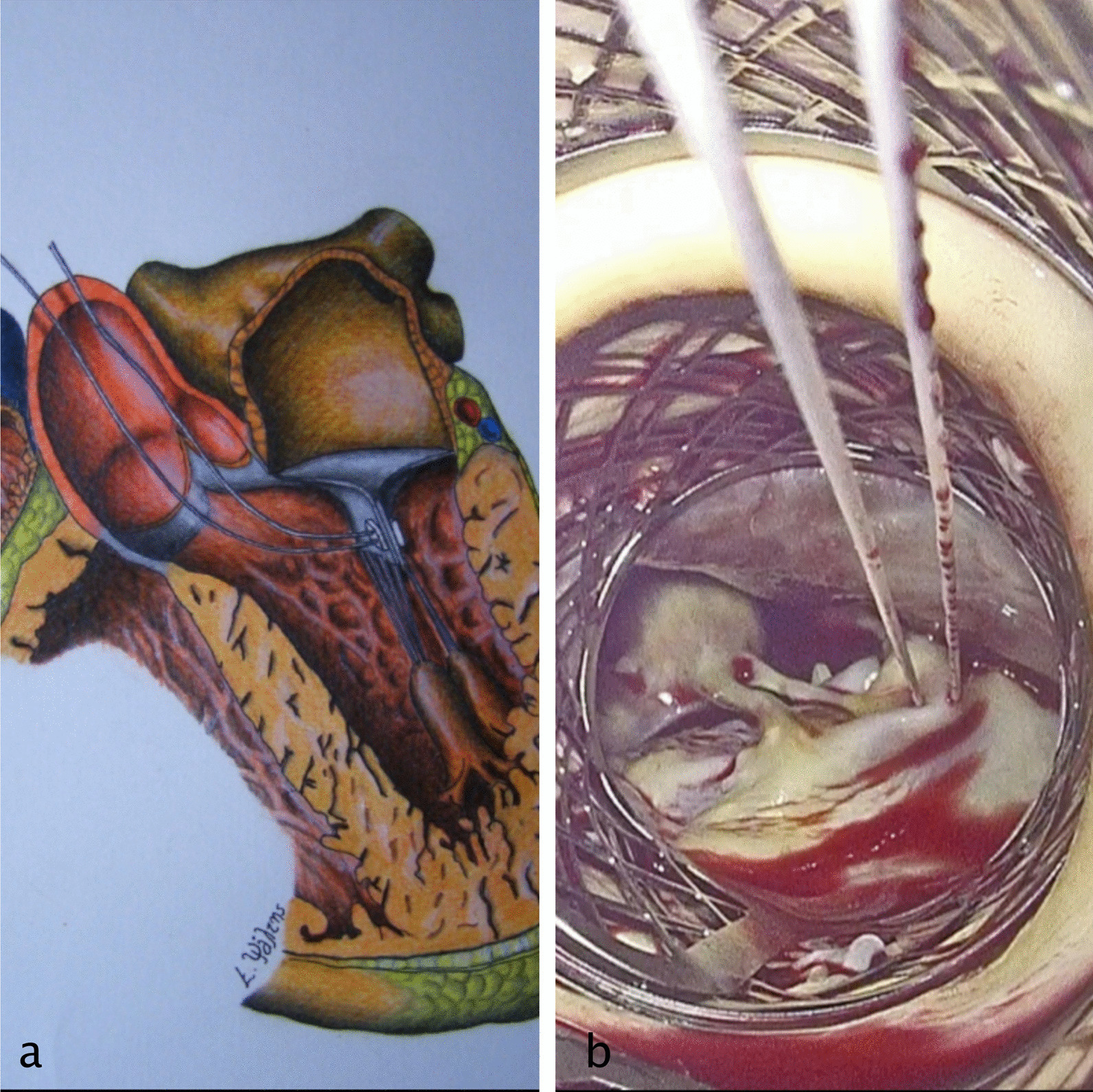
A successful surgical approach of TEAVR and concomitant trans-aortic mitral valve repair using the edge-to-edge technique for secondary mitral regurgitation: **a** schematic drawing and **b** endoscopic image

**Fig. 2:**
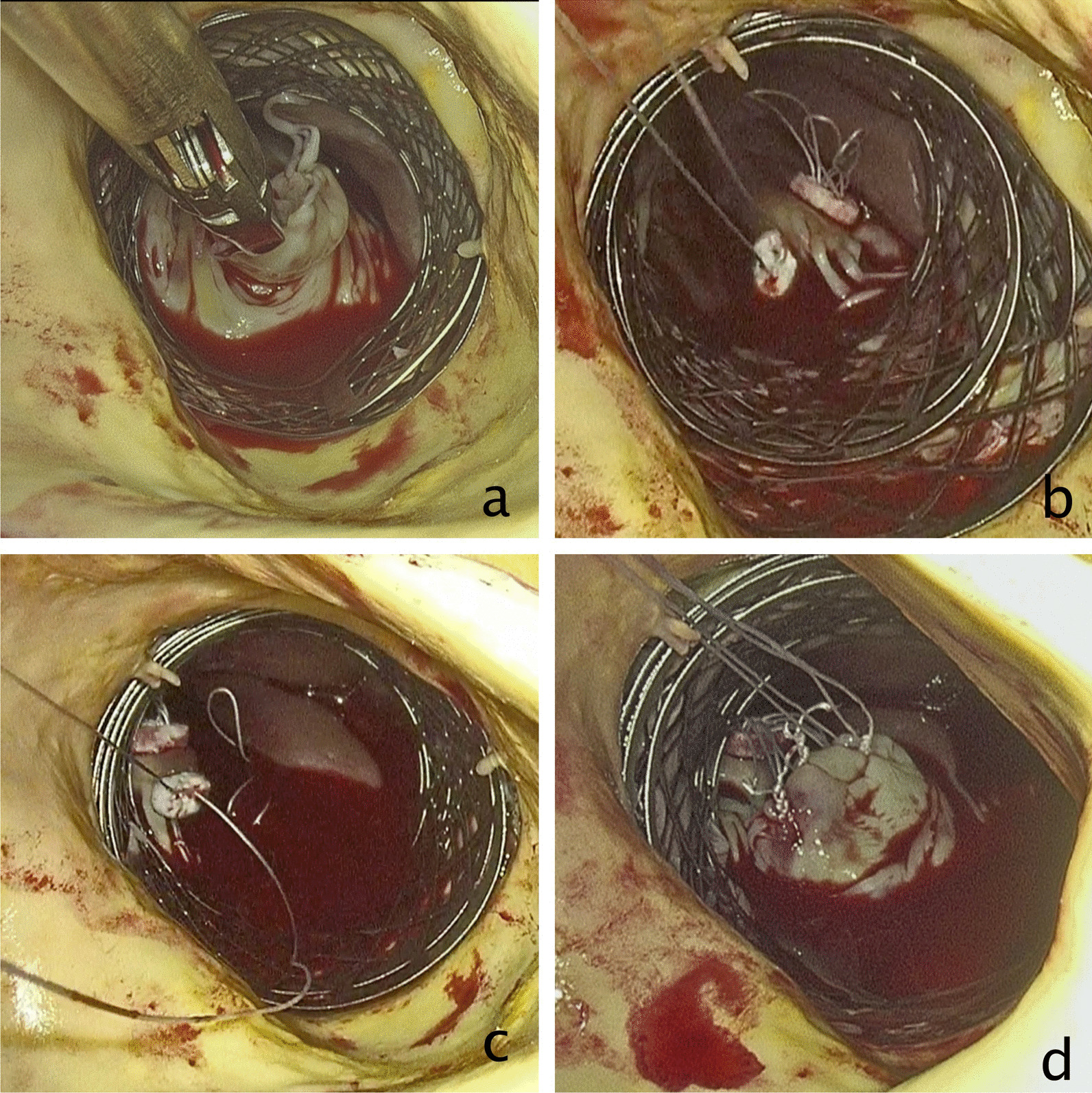
Totally endoscopic trans-aortic mitral repair with 4 neochords to P2 for a flail P2, in combination with TEAVR: **a** P2 flail, **b** suturing a set of 4 loops size 16 mm to the head of the posteromedial papillary muscle, **c** suturing a set of 4 loops size 16 mm to the head of the anterolateral papillary muscle, **d** suturing the free edge of 4 loops (2 from the anterolateral and 2 from the posteromedial papillary muscles) to the free edge of the flail P2

In cases of secondary MR, a horizontal mattress, teflon buttressed PTFE suture is used to perform an edge-to-edge repair between A2 and P2 using a deep bite of at least 5 to 7 mm (see Fig. 1a and b, and Additional file 2: Video S2). In cases of primary MR, a second metal net is inserted inside the first one and at the level of the anterior mitral leaflet (see Fig. 2a and Additional file 1: Video S1). A prefabricated set of the pre-measured length of PTFE neochords (Seramon chordae loop, Serag Wiessner, DE) are inserted and secured at the fibrous head of the corresponding papillary muscle (Fig. 2b and c). The open end of the loops (neochords) are then secured to the free edge of the prolapsing/flail segment of the mitral leaflet (Fig. 2d). Several loops 4 to 5 mm apart in the prolapsing segments are used in order to spread the leaflet tension and to improve the durability of the repair. This type of repair is only applicable for anterior leaflet prolapse and middle scallop (P2) posterior leaflet prolapse. Prolapse/flail of the anterolateral (P1) and posteromedial (P3) scallops, and of the two commissures, cannot be repaired with the trans-aortic approach, because these segments are well hidden by the anterior leaflet and its chords. Also marked dilation of the mitral annulus is a relative contraindication of this technique (better managed with an atriotomy in order to perform an annuloplasty). Alternatively, the loop technique with the edge-to-edge approach can be combined to achieve a watertight mitral valve through the trans-aortic approach, but so far, we have not performed this type of repair.

Finally, we proceed with the aortic valve replacement with a conventional mechanical or biological prostheses, as previously described by our group [1]. XC and CPB times for the combined approach is reasonable (below 90 and 120 min respectively for primary MR and below 60 and 90 min respectively for secondary MR, after the initial learning curve of the technique). Short term follow-up (mean 5.7 months, range: 0.93–9.9) of our 13 patients operated with the described technique have been excellent with MR ≤ 1+.

## Discussion and conclusion

Totally endoscopic approaches will increasingly become more popular in the years to come. A successful surgical technique of TEAVR and concomitant totally endoscopic mitral valve repair via a trans-aortic approach is presented. When stand-alone endoscopic mitral repair is performed, this is usually done through a more lateral thoracotomy incision through the 4th ICS [2]. In order to perform a combined aortic valve replacement and a mitral repair through a standard left atriotomy incision, a working incision is placed through the 3rd ICS in the middle clavicular line, which is a compromise for both the endoscopic aortic valve replacement and the mitral repair through a left atriotomy. Such an approach makes the placement of the aortic annular sutures more difficult due to the fact that the working incision is not aligned with the aortic annulus, this will prolong the XC time in excess of 120 min (mins) and the CPB time in excess of 180 min. The aortotomy mitral repair approach presented here significantly shortens the XC and CPB times to 90 and 120 min, respectively for primary MR, and to 60 and 90 min, respectively for secondary MR, thus making it possible to perform double valve (aortic and mitral) totally endoscopic surgery within reasonable XC and CPB times that are comparable to median sternotomy approaches.

Contraindications of the presented technique are:

- marked mitral annular dilation—better managed with an annuloplasty procedure through a left atriotomy [4] and
- P1 or P3 or commissural flail/prolapse—better managed with the loop technique through a left atriotomy [2, 4] (through a median sternotomy incision if combined with aortic valve disease).

Also, contraindications of the totally endoscopic approach through an aortotomy incision in general are:

iii) heavily calcified ascending aorta,
iv) obesity and
v) inability to go on CPB through the femoral vessels.

The presented method substantially decreases intensive care times and hospital length-of-stay, and allows patients to return to work sooner. It should be mentioned that the need for simultaneous aortic and mitral valve surgery is not uncommon, since significant mitral regurgitation can be found in patients with bicuspid aortic valve and coronary artery disease [5].

## Supplementary Information

**Additional file 1. Video S1**. A case of severe aortic stenosis and severe mitral regurgitation due to a flail P2; the loop technique (4 neochords to P2) was used trans-aortically with a totally endoscopic approach.

**Additional file 2. Video S2**. A case of severe aortic stenosis and severe mitral regurgitation due to a dilated cardiomyopathy and tethering of both the anterior and posterior leaflets; the edge-to-edge technique was used trans-aortically with a totally endoscopic approach.

## Data Availability

The datasets used and/or analysed during the current study are available from the corresponding author on request.

## Abbreviations

TEAVR: Totally endoscopic aortic valve replacement
SAVR: Surgical aortic valve replacement
XC: Cross clamp
CPB: Cardiopulmonary bypass
MR: Mitral regurgitation
TEE: Transesophageal echocardiography
ICS: Intercostal space
